# Role of confirmed and potential predictors of an unfavorable outcome in heart failure in everyday clinical practice

**DOI:** 10.1007/s11845-020-02477-z

**Published:** 2021-02-17

**Authors:** Anna Chuda, Maciej Banach, Marek Maciejewski, Agata Bielecka-Dabrowa

**Affiliations:** 1 Heart Failure Unit, Department of Cardiology and Congenital Diseases of Adults, Polish Mother’s Memorial Hospital Research Institute, Rzgowska 281/289, 93-338 Lodz, Poland; 2 Department of Hypertension, Chair of Nephrology and Hypertension, Medical University of Lodz, Zeromskiego 113, 90-549 Lodz, Poland; 3 Department of Cardiology and Congenital Diseases of Adults, Polish Mother’s Memorial Hospital Research Institute, Rzgowska 281/289, 93-338 Lodz, Poland

**Keywords:** Biomarker(s), Heart failure, Prognosis, Risk factor(s), Risk models, Risk prediction

## Abstract

Heart failure (HF) is the only cardiovascular disease with an ever increasing incidence. HF, through reduced functional capacity, frequent exacerbations of disease, and repeated hospitalizations, results in poorer quality of life, decreased work productivity, and significantly increased costs of the public health system. The main challenge in the treatment of HF is the availability of reliable prognostic models that would allow patients and doctors to develop realistic expectations about the prognosis and to choose the appropriate therapy and monitoring method. At this moment, there is a lack of universal parameters or scales on the basis of which we could easily capture the moment of deterioration of HF patients’ condition. Hence, it is crucial to identify such factors which at the same time will be widely available, cheap, and easy to use. We can find many studies showing different predictors of unfavorable outcome in HF patients: thorough assessment with echocardiography imaging, exercise testing (e.g., 6-min walk test, cardiopulmonary exercise testing), and biomarkers (e.g., N-terminal pro-brain type natriuretic peptide, high-sensitivity troponin T, galectin-3, high-sensitivity C-reactive protein). Some of them are very promising, but more research is needed to create a specific panel on the basis of which we will be able to assess HF patients. At this moment despite identification of many markers of adverse outcomes, clinical decision-making in HF is still predominantly based on a few basic parameters, such as the presence of HF symptoms (NYHA class), left ventricular ejection fraction, and QRS complex duration and morphology.

## Heart failure in numbers

Heart failure (HF) is a cardiovascular disease with an ever increasing incidence [1]. In the National Health and Nutrition Examination Survey (NHANES) data in USA, in 6.2 million Americans, HF was diagnosed in the period 2013–2016 compared with 5.7 million in the period 2009–2012 [2]. This disease affects an estimated 26 million people worldwide, including 1–2% of the adult population of developed countries in America and Europe, and as many as 10% in people over 70 years [1–8]. The prevalence of HF is estimated to be about 20/1000 people, and as high as 130/1000 people for those aged over 65 years [1–8], which results in more than 1 million hospitalizations annually in both the USA and Europe. About 15 million people suffer from it in the whole of Europe. In Western Europe, there are over 5 million HF patients [1, 3, 4], and in Poland nearly 1 million (about 3% of the population). Another 10 million Poles are at risk of this disease—mainly people with hypertension, coronary artery disease, obesity, diabetes, and smoking cigarettes [9–14]. In the USA, there are around 5 million HF sufferers. About 400,000 new cases of HF are diagnosed in the USA annually [2, 5]. The number of new cases of HF reported each year in Europe is approximately 2–3/1000. Among the 70–80 age group, 100/1000 people have HF every year [1, 3, 4]. By 2030, the number of HF patients will increase by half [1]. For example, in the USA, the number of HF patients will exceed 8 million people [2, 5]. By the year 2050, a quarter of the population will be older than 65 years of age in developed countries [1]. In 1950, in Europe, the average age of the population was 29.2 years, and by 1998, this had risen to 37.1 years. By 2050, the average age of the population is expected to be 47.7 years, leading to a higher prevalence of HF [15]. HF is the main cause of death worldwide [16]. Annual morbidity of HF in developed countries is 5–10 people per 1000 inhabitants [1, 3, 4]. HF is associated with high consumption of healthcare resources [4, 7]. This results in high costs of care for a patient with HF, which mostly results from repeated hospitalizations [4, 7]. HF, through reduced functional capacity, frequent exacerbations of disease, and repeated hospitalizations, results in poorer quality of life, decreased work productivity, and significantly increased costs of the public health system [16].

## The importance of predictors of heart failure course

The main challenge in the treatment of HF is the availability of reliable prognostic models that would allow patients and doctors to develop realistic expectations about the prognosis and to choose the appropriate therapy and monitoring method. Prognosis assessment plays a special role in patients qualified for implantable device therapy or surgical treatment (including heart transplantation). Prognosis also plays an important role in planning terminal palliative care with the patient and his family. Not only does the predictor allow one to identify a high-risk patient in advance but it also allows one to monitor and implement individual preventive therapy. Secondly, identifying factors that contribute to poor prognosis can help develop new, targeted therapies [17]. This article begins with a review of individual markers that contribute to the risk of unfavorable outcome in HF.

## Characteristics of clinically useful prognostic factors

Predictors should be easily obtainable and associated with some therapeutic and clinical results [17].

The 2016 European Society of Cardiology (ESC) guidelines on HF named over 70 predictors in HF patients [8]. A modified list is presented in Table 1.

**Table 1:** Markers of unfavorable outcome in HF (according to [8], modified)

Demographic data	Older age, male sex, low socio-economic status
Medical history	Ischemic etiology, longer HF duration, previous HF hospitalization, adequate and inadequate high-energy ICD interventions, non-compliance with evidence-based HF therapies (β-blockers, RAAS inhibitors)
Clinical status	Advanced NYHA class, high resting heart rate, low SBP, clinical signs of volume overload (e.g., pulmonary congestion, peripheral edema, jugular vein dilatation, hepatomegaly) and of peripheral hypoperfusion, Cheyne-Stoke ventilation, lower BMI, frailty
Cardiac imaging, including echocardiography	LV systolic dysfunction (low LVEF, reduced GLS), LV dilatation, LV hypertrophy, severe LV diastolic dysfunction, pseudonormal/restrictive LV filling pattern, left atrial dilatation, pulmonary hypertension, right ventricle dilatation and dysfunction, dyssynchrony, severe valvular disease, large territory of non-viable myocardium or of inducible ischemia in imaging stress testing, late gadolinium enhancement in CMR
Electrocardiogram	Wide QRS complex, ventricular arrhythmia, atrial fibrillation
Exercise testing	Short 6-min walk test distance, reduced VO_2_peak and high VE/VCO_2_slope in cardiopulmonary exercise test
Genetic testing	Lamin A/C—LMNA mutations (especially non-missense mutations), phospholamban (PLN) mutation
Non-cardiac comorbidities	Previous stroke/TIA, peripheral artery disease, diabetes, anemia, iron deficiency, COPD, sleep apnea (both central and obstructive), kidney/liver dysfunction, depression

*Abbreviations: BMI*, body mass index; *BUN*, blood urea nitrogen; *CMR*, cardiac magnetic resonance; *COPD*, chronic obstructive pulmonary disease; *eGFR*, estimated glomerular filtration rate; *HF*, heart failure; *ICD*, implantable cardioverter-defibrillator; *LV*, left ventricle; *LVEF*, left ventricular ejection fraction; *NYHA*, New York Heart Association; *RAAS*, renin-angiotensin-aldosterone system; *RNA*, ribonucleic acid; *SBP*, systolic blood pressure; *TIA*, transient ischemic attack; *VE/VCO*_*2*_, minute ventilation/carbon dioxide production; *VO*_*2*_*peak*, peak oxygen uptake; *WBC*, white blood cell count

The 2019 ACC Expert Consensus Decision Pathway on Risk Assessment, Management, and Clinical Trajectory of Patients Hospitalized With Heart Failure also named many predictors of unfavorable outcome during hospitalization in HF patients [18]. A modified list is presented in Table 2.

**Table 2:** Risk factors during hospitalization in HF (according to [18], modified)

**Assessment prior to admission**	
**▪** Older age **▪** Number of previous HF hospitalizations **▪** Comorbidities, especially diabetes, COPD, liver disease, cancer, dementia **▪** Frailty **▪** Known low LVEF in HFrEF **▪** RV dysfunction	
**Assessment at admission**	**Reassessment at discharge**
NYHA Class IV symptoms	Effective decongestion
Nonadherence to medications or salt/fluid restriction	Adherence
Elevated natriuretic peptide (NP) levels on admission	% reduction (> 30–60%) in NP levels Discharge NP levels
Elevated serum creatinine or low clearance on admission	Small increases in creatinine accompanying successful decongestion
High BUN on admission	High BUN at discharge
Low spot urine sodium after first IV diuretic dose	Low total urinary sodium excretion Total urine output during hospitalization
Diuretic resistance with high outpatient doses	Diuretic resistance in-hospital High loop diuretic doses at discharge
Degree of congestion at admission	Residual congestion after treatment **▪** High measured filling pressures **▪** Orthopnea **▪** Edema **▪** Composite congestion scores **▪** Lack of hemoconcentration
Hemodynamic profile of “cold and wet” at admission	Discharge with either “cold” or “wet” profile
Low systolic blood pressure	Low systolic blood pressure at discharge
Troponin elevation	Troponin elevation at any time during hospitalization
Hyponatremia	Lower sodium at discharge
**▪** No RAS therapy **▪** No beta blocker therapy	Discontinuation of ACEI/ARB in hospital for hypotension or kidney dysfunction Discharge without RAS inhibition or discharge without beta-blocker
**Unexpected in-hospital events conferring additional risks**	
**▪** Resuscitation or intubation **▪** Intravenous inotropic therapy even if brief	

Abbreviations: *HF* – heart failure; *COPD* — chronic obstructive pulmonary disease; *LVEF* — left ventricular ejection fraction; *HFrEF* — heart failure with reduced ejection fraction; *RV* — right ventricle; *NYHA* — New York Heart Association; *BUN* — blood urea nitrogen; *IV* — intravenous; *RAS* — Renin-Angiotensin System; *ACEI* - Angiotensin converting enzyme inhibitors; *ARB* - Angiotensin II receptor blockers

However, no single risk factor is sufficient to predict prognosis in HF. Results of a few markers must be interpreted together. Still it is important to find the most important and valuable panel of a few predictors and there are still ongoing studies assessing potential new ones.

## Conversation with the patient—still important

Knowledge of a patient’s demographic, medical, and clinical data could play an important role in prediction of life expectancy. Previous studies have shown that male sex is more strongly associated with left ventricular systolic dysfunction, but female sex is more strongly associated with preserved left ventricular function [19–21]. Ischemic etiology and coronary heart disease are strongly correlated with male sex [19–21]. Pathophysiological mechanisms that could explain sex-related differences can be separated into differences in bio-hormonal system activity (inflammation, oxidative stress, sympathetic nervous system, hormonal system), various cardiovascular risk factors, and various comorbidities (coronary artery disease, atrial fibrillation, hypertension, obesity, and diabetes and/or insulin resistance) [19–21]. These differences can influence mortality and morbidity differences between genders [21]. In LaMarca et al. study, animal models have shown that the sex-specific mitochondrial adaptation to effort is modulated by the estrogen receptor ERβ [22]. In the failing heart, sexual differences have been identified in the expression of genes involved in energy metabolism. Female pattern involves genes related to energy metabolism and regulation of transcription and translation while the male pattern involves genes related to muscular contraction. Failed female hearts maintain energy metabolism better than male hearts and are better protected against calcium overload. As a result, female sex can be protective against HF mortality [22].

Low socioeconomic status in adulthood and childhood is associated with worsened HF outcomes [23–25]. Low socioeconomic status in childhood is associated with worse HF risk factors in adulthood, such as smoking [26, 27], high blood pressure [28–30], obesity [31–33], and coronary heart disease [34, 35].

## Physical examination

In the general population, increased systolic blood pressure (SBP) is associated with unfavorable outcomes and higher risk of development of HF. In the Framingham Heart Study population, 91% of the participants with HF had a previous diagnosis of hypertension [6]. Compared with the normotensive individuals, patients with higher SBP had 2- and 3-fold increased risk of developing HF [2]. However, in patients with heart failure with reduced ejection fraction (HFrEF), high SBP is associated with better outcomes. SBP has a U-shaped association with mortality in patients with 30%≤ LVEF< 50% and a linear association with mortality in patients with LVEF< 30%. As a result, lower SBP is associated with increased mortality in HFrEF patients [36].

In the general population, increased body mass index (BMI) predisposes to development of HF (5% increase in risk for each 1 kg/m^2^ increase in BMI) [37, 38]. In the Mahajan et al. meta-analysis, intentional weight loss in obese patients without HF was associated with a reduction in left atrial size (*p* = 0.02), a reduction in left ventricular mass index (*p* < 0.0001), and improvement in left ventricular diastolic function (*p* ≤ 0.0001) [39]. However, in the HF population, higher BMI is associated with lower risk of worsened outcomes—a 10% reduction in mortality for each 5-unit increase in BMI was observed in the Kenchaiah et al. [40] and Fonarow et al. studies [41]. In the Mahajan et al. meta-analysis, the “obesity paradox” was also observed for all-cause mortality, and for cardiovascular (CV) mortality in the overweight group (OR = 0.86 (95% CI 0.79 to 0.94), *n* = 11) [42].

The 2016 guidelines of the ESC identified cachexia and sarcopenia as important comorbidities of HF [8]. Cachexia (loss of body weight) develops in the course of disease in the catabolic stage. The cachectic patient may lose any type of tissue, leading to weight loss [43]. Cardiac cachexia has been observed as an independent risk factor of death in patients with HF [44, 45]. Sarcopenia (skeletal muscle wasting) is an important comorbid disease. In the Morley et al. study, sarcopenia was observed in 19.5% of all HFrEF patients [46]. The Bekfani et al. study with heart failure with preserved ejection fraction (HFpEF) patients confirmed a similar prevalence [47]. Reduced lean mass (LM) was independently associated with abnormal cardiorespiratory function and muscle strength, leading to worse prognosis and reduced quality of life in HF patients [44]. A multicenter Italian study identified sarcopenia as an important factor for prolonged hospitalization in HF patients admitted to acute care wards (5.1 days vs. 3.2 days) [48]. Many studies have also demonstrated that the loss of skeletal muscle mass is associated with loss of physical independence and, as a result, with significantly worsened prognosis and an increased risk of death in HF patients [43, 44, 46–50].

The New York Heart Association (NYHA) functional classification is still useful for assessing syndrome severity, patient’s exercise tolerance and prognosis in HF patients [51, 52]. NYHA functional class correlates with the magnitude of signs of cardiovascular impairment in these patients and has been associated with mortality in HF [51–54].

A list of other significant values from the medical history and clinical status of the patient is presented in Tables 1 and 2.

## Echocardiographic imaging

Echocardiography provides detailed information regarding cardiac structure and function [8, 17]. HFrEF can be easily diagnosed by echocardiography and is understood as left ventricular ejection fraction (LVEF) < 40% [8]. Diagnostic criteria for HFpEF have been far more problematic so far. In 2019, a writing committee initiated by the HFA of the ESC therefore produced an updated consensus recommendation—the HFA–PEFF diagnostic algorithm [55]. A modified version is presented in Table 3.

**Table 3:**
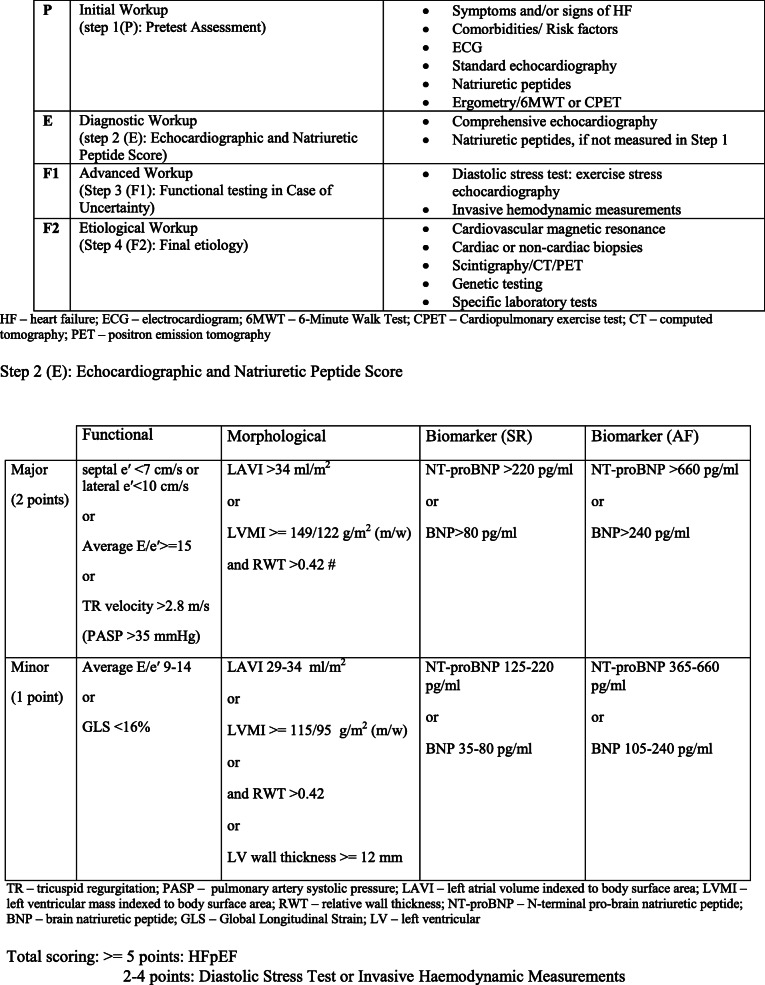
HFA-PEFF diagnostic algorithm (according to [55], modified)

The CHARM trial [56] showed that each 10% reduction in EF was associated with a 39% increase in the risk of mortality, but this was only for EF below 45% [56]. Many measurements of structure and function of the cardiovascular system correlate with mortality in HF.

**Fig. 1:**
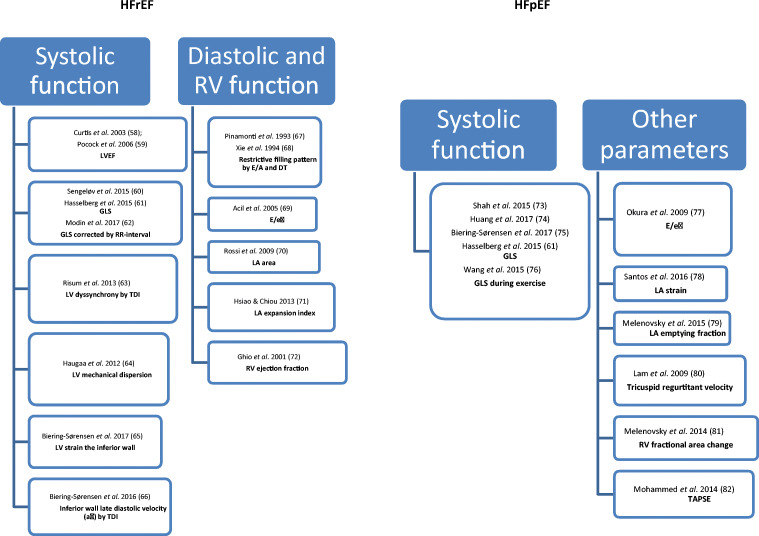
Pathophysiological interplay between AF-HF cycle and HF–AF cycle (according to [18], modified). Abbreviations: DT, deceleration time of the E-wave; GLS, global longitudinal strain; LA, left atrial; LV, left ventricular; LVEF, left ventricular ejection fraction; RV, right ventricle; TAPSE, tricuspid annular plane systolic excursion; TDI, tissue Doppler imaging

Many echocardiographic markers have prognostic value in HF (Fig. 1; Tables 1, 2, and 3).

### New parameters with prognostic value in HFrEF

Mechanical dyssynchrony (the late diastolic velocity (a′)) measured by tissue Doppler imaging (TDI) and LV dyssynchrony based on global longitudinal strain (GLS) imaging seem to be important prognostic markers in HFrEF [60]. Localized areas with a changed cardiac structure (such as scarring, fibrosis, ischemia) can be missed by a global measure, such as the LVEF [57]. For example, in patients with ischemic cardiomyopathy receiving ICD, only a′ measured by TDI in the inferior wall is a predictor of VT/VF and cardiovascular death [66].

In atrial fibrillation (AF) rhythm, the varying RR interval and changing loading conditions make it difficult to measure LV systolic function. A new method of correcting GLS by the RR seems to be an important marker for evaluation of LV systolic function in HFrEF patients with AF [62, 83].

Left atrium (LA) volumes and function were the best echocardiographic markers of clinical outcomes (mortality and hospitalization) [70], as a sensitive barometer of LV filling pressure [84, 85]. The power of LA parameters measured with the LA emptying fraction and the LA expansion index (LAEI) could be useful. In the Hsiao study [71], LAEI was better compared to LA volume in predicting death and hospitalization for HF.

However, no single risk factor is sufficient to determine prognosis in HFrEF patients. The value of a few echocardiographic parameters must be interpreted together (systolic, diastolic, and RV function). Figure 1 shows many echocardiographic predictors of outcome in HFrEF (Fig. 1).

### New parameters with prognostic value in HFpEF

LVEF may be correct in HFpEF, even though systolic dysfunction is already appearing. The abnormal LV contraction includes abnormal longitudinal shortening (results from dysfunctional or stressed longitudinal myofibres; measured by impaired mitral annular plane longitudinal descent and velocity, decreased GLS) [86, 87], preserved or increased circumferential shortening (measured by circumferential strain—CS; results from subendocardial fiber dysfunction with left-handed helix shortening by unbalanced subepicardial fibers) [57, 88], and increased wall thickness-to-chamber radius ratio (results from concentric hypertrophy; radial thickening; measured by radial strain—RS).

Mitral annular plane systolic excursion (MAPSE) has been suggested as a parameter for impaired longitudinal function and could provide complementary information to EF [86].

GLS measured during bicycle ergometer testing has also been identified as an important prognostic marker in HFpEF [76].

As already mentioned before, LA volumes and function are sensitive indicators of LV filling pressure [84]. Strain imaging by 2D speckle-tracking (2DS) is a new index of LA function. Recent data demonstrated that LA strain is decreased in diastolic HF [89]. This new parameter could be useful in categorizing diastolic dysfunction [89] and may have prognostic value in HFpEF [78].

Greater right ventricular (RV) afterload results in pulmonary hypertension (measured by tricuspid regurgitation (TR) velocity) and RV systolic dysfunction (measured by TAPSE) is highly prevalent in HFpEF [80]. 2DS RV free wall strain may have prognostic value in HFpEF, despite the complicated geometry of the RV [57].

In summary, LV systolic and diastolic function, LA function, and RV function have prognostic value in HFpEF. Figure 1 provides a list of studies that have identified many echocardiographic prognostic parameters in HFpEF (Fig. 1).

## Exercise testing in HF

### Six-min walk test

The 6-min walk test is useful in measuring functional limitation (patient’s exercise capacity) in the prognostic stratification and in evaluating the effects of therapy in children and adults with HF [90, 91]. Hsich et al. [92] observed in one study a 7% increase in mortality for each 1-min reduction in exercise capacity in HF patients. The SOLVD study [93] showed that 6MWT distance was an important and independent predictor of morbidity (heart failure hospitalization) and mortality in a logistic regression model in patients with left ventricular dysfunction. In the studies by Rostagno et al. [94], Cahalin et al. [95], and Arslan et al. [96], lower functional capacity (distance of ≤ 300 m in 6MWT) was a useful prognostic marker of death or hospitalization in patients with mild-to-moderate heart failure. The Ingle et al. study [97] also showed that the 6MWT distance is an important independent predictor of all-cause mortality in patients with HF. In the study by Boer et al. [98], 6MWT distance was a simple and feasible tool to identify children with a higher risk of death or heart transplantation in children with dilated cardiomyopathy. However, there are no data showing the prognostic usefulness of 6MWT in women, in elderly patients, or in patients with left ventricular diastolic dysfunction [99].

**Fig. 2:**
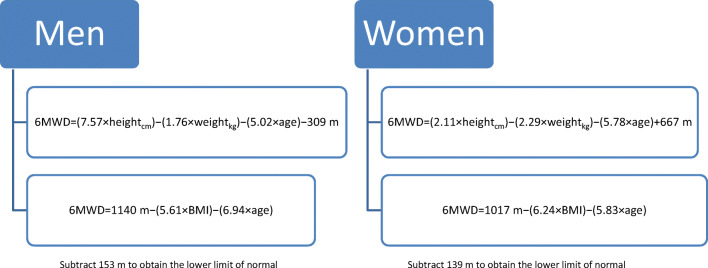
Reference value for the 6MWT distance corrected by anthropometric variables in a group of healthy subjects (according to [99, 104], modified). Abbreviations: BMI, body mass index

6MWT poorly correlates with hemodynamic and functional echocardiographic parameters [99]. In the Zugck et al. [100] and Opasich et al. [101] studies, only right ventricular ejection fraction correlated significantly with 6MWT distance. However, distance walked during 6MWT correlated significantly with non-cardiovascular parameters (muscular strength, postural balance, reaction time, mood, and general health) [99] and with demographic variables, such as gender (lower in women), weight and age (negative correlation) [102], and height (positive correlation) [99]. This suggests that the test result should be evaluated not only as a total distance walked in meters but also as a percentage of the predicted value (6MWD%) [103]. Figure 2 provides the reference value for the 6MWT distance corrected by anthropometric variables in a group of healthy subjects (Fig. 2).

6MWT distance can also be used to evaluate the effect of therapeutic interventions in patients with HF (current pharmacological therapy, program of physical training, new drugs in addition to standard therapy, ventricular assistance devices, ventricular resynchronization techniques) [99]. However, the correct total distance, percentage change from baseline, or percentage change of predicted value is not specified yet [99].

### Cardiopulmonary exercise testing

Cardiopulmonary exercise testing (CPET) through measurement of peak oxygen uptake (VO_2_) defines maximum exercise capacity of the patient. CPET evaluation should start with maximum effort (RER > 1.0 to 1.1) [105]. Peak VO_2_ remains the gold standard in predicting outcome in HF. Peak VO_2_ < 14 ml/kg/min and < 12 ml/kg/min in patients on β-blockers, in the HFrEF population continues to be a significant prognostic factor. It is also an important predictor of death in HFpEF patients [106, 107]. For young, obese, and cachectic patients, peak VO_2_ should be interpreted as a percentage of predicted, with values < 50% indicating a poor prognosis [108]. Peak VO_2_ on effort and anaerobic threshold (AT) were used to determine the classification of the severity of HF (Table 4). For patients who do not make enough effort, oxygen uptake efficiency slope (OUES) < 1.47 l/min and VO_2_ at VT < 9 ml/kg/min indicate a bad prognosis [110]. Mean response time (MRT), a sensitive indicator of O_2_ uptake kinetics, more accurately showed the ability to increase cardiac output during low-level exercise. An MRT > 60 s correlated with a decrease in exercise right ventricular ejection fraction (RVEF) and a decrease in cardiac output (through the increased transpulmonary gradient).

**Table 4:** Classification of the severity of HF depending on the CPET result (according to [109], modified)

Class	Severity of HF	VO_2_ peak (ml/kg/min)	VO_2_-AT (ml/kg/min)
A	Mild/none	> 20	> 14
B	Mild/moderate	16–20	11–14
C	Moderate/severe	10–16	8–11
D	Severe	6–10	5–8
E	Very severe	< 6	< 4

Failure to achieve SBP > 120 mm Hg and no increase in SBP during exercise are associated with poor prognosis [111]. Both chronotropic incompetence and slow return to normal heart rate in recovery (< 6 bpm) also indicate poor prognosis [112].

VE/VCO_2_ slope and exercise oscillatory ventilation (EOV) are among the strongest predictors of poor outcome in HF. VE/VCO_2_ slope > 34 to 36 indicates high-risk HF patients [113]. The presence of EOV is consistently associated with an annual mortality of > 20% [114].

In summary, peak oxygen uptake parameters, ventilatory efficiency or stability, and chronotropic incompetence are the main CPET factors in predicting outcome in HF patients.

CPET is not often used in chronic HF patients for a few reasons, including the limited availability of equipment (relatively expensive technology, expertise required) and patient’s inability to perform a maximal effort test (comorbidities, cognitive impairment) [99, 115]. Alternatively, severity of inspiratory muscle weakness (IMW), measured by maximal static inspiratory pressure (PImax) could be a cheap and useful parameter of inspiratory muscle strength as a marker of maximal work of breathing and can represent a reasonable alternative to pVO_2_ for mortality risk stratification in HF patients [115]. In previous studies [115–118], PImax was a strong and independent predictor of mortality in HFrEF patients, especially in individuals unable to perform an exercise test.

## Laboratory biomarkers

Multiple impaired regulatory axes are seen in HF, including the renin-angiotensin-aldosterone system (RAAS), sympathetic regulation, neurohormonal regulation, and the cardiac stretch response. HF is associated with a chronic inflammatory state, oxidative stress, and in effect extracellular matrix remodeling [119]. Many prognostic biomarkers in HF have been identified. A classification of useful biomarkers based on their pathophysiological role in HF is presented in Table 5.

**Table 5:** Classification of biomarkers based on their pathophysiological role in HF (according to [120], modified)

Pathophysiological pathway	Biomarkers
Myocyte stress	BNP; NTpro-BNP; NTpro-ANP; MR-proADM; sST2
Myocyte injury	TnT; TnI; CK-MB mass; MLCK-I; hFABP; PTX3; HSPs
Inflammation	hsCRP; TNF-α; sTNFR; cytokines (e.g. IL-1, IL-6, IL-18); AdipoQ, sST2; PTX3; OPG; PCT
Oxidative stress	oxLDL; MPO; urinary biopyrrins; IsoPs; MDA
Neurohormones	NE; renin; AngII; aldosterone; AVP/copeptin; EDNs; Cg; ADM; MR-proADM
Extracellular matrix remodeling	MMPs; TIMPs; P1NP; P3NP; Gal-3; sST2; GDF-15
Cardio-renal syndrome	Serum creatinine; ACR; CysC; NGAL; BTP
Others	Hbg; serum albumin; RDW, VCAM

Abbreviations: *BNP*, brain natriuretic peptide; *NTpro-BNP*, N-terminal pro-brain natriuretic peptide; *NTpro-ANP*, N-terminal proatrial natriuretic peptide; *MR-proADM*, mid-regional pro-adrenomedullin; *sST2*, soluble ST2; *TnT*, troponin T; *TnI*, troponin I; *CK-MB mass*, creatine kinase myocardial band fraction; *MLCK-I*, myosin light-chain kinase I; *hFABP*, heart-type fatty acid binding protein; *PTX3*, pentraxin-related protein; *HSPs*, heat shock proteins; *hsCRP*, high-sensitivity C-reactive protein; *TNF-α*, tumor necrosis factor α; *sTNFR*, soluble tumor necrosis factor receptors; *IL-1*, interleukin 1; *IL-6*, interleukin 6; *IL-18*, interleukin 18; *AdipoQ*, adiponectin; *OPG*, osteoprotegerin; *PCT*, procalcitonin; *oxLDL*, oxidized low-density lipoprotein; *MPO*, myeloperoxidase; *IsoPs*, isoprostanes; *MDA*, plasma malondialdehyde; *NE*, norepinephrine; *AngII*, angiotensin II; *AVP*, arginine vasopressin; *EDNs*, endothelins; *Cg*, chromogranins; *ADM*, adrenomedullin; *MMPs*, matrix metalloproteinases; *TIMPs*, tissue inhibitors of metalloproteinases; *P1NP*, procollagen type 1 N propeptide; *P3NP*, procollagen type 3 N propeptide; *Gal-3*, galectin 3; *GDF-15*, growth/differentiation factor 15; *ACR*, urine albumin to creatinine ratio; *CysC*, cystatin C; *NGAL*, neutrophil gelatinase-associated lipocalin; *BTP*, β-trace protein; *Hbg*, hemoglobin; *RDW*, red blood cell distribution width; *VCAM*, vascular cell adhesion molecule

Nowadays, natriuretic peptides are the gold standard biomarkers [8]. Brain natriuretic peptide has been shown to predict morbidity, mortality, and hospitalization from HF in clinical practice [121–123]. In the trial (PROTECT) by Januzzi et al. [124], patients who had NT-proBNP-guided therapy for heart failure benefited. Each natriuretic peptide has specific cut-off concentrations (Table 6). Plasma concentrations should be interpreted in the context of the clinical setting of the patient [125].

**Table 6:** Recommended natriuretic peptide cut-offs for HF diagnosis (according to [125], modified)

Cut-off levels (pg/ml)						
	NT-proBNP			BNP		
	Age < 50	Age 50–75	Age > 75	Age < 50	Age 50–75	Age > 75
Acute setting, patient with acute dyspnea						
HF unlikely	< 300			< 100		
“Gray zone”	300–450	300–900	300–1800	100–400		
HF likely	> 450	> 900	> 1800	> 400		
Non-acute setting, patient with mild symptoms						
HF unlikely	< 125			< 35		
“Gray zone”	125–600			35–150		
HF likely	> 600			> 150		

Abbreviations: *BNP*, B-type natriuretic peptide; *HF*, heart failure; *NT-proBNP*, N-terminal proBNP

Consider reducing the cut-off levels in obese patients by 50%

Previous studies have shown different biomarker profiles between patients with HFrEF, heart failure with mid-range ejection fraction (HFmrEF), and HFpEF. For example, in the Tromp et al. study [126], HFmrEF was associated with hemoglobin, red blood cells, BNP, galectin-3, endothelin-1, and syndecan-1. In contrast, HFrEF was mostly associated with BNP, kidney injury molecule-1 (KIM-1), troponin-I (TnI), red blood cells, and hemoglobin, whereas HFpEF was associated with BNP, angiogenin, hemoglobin, galectin-3, D-dimer, and inflammation markers (pentraxin-3, RAGE) and a remodeling marker (osteopontin). In another Tromp et al. study [127], the main proteins in HFrEF were NTproBNP, growth differentiation factor-15 (GDF-15), interleukin-1 receptor type 1 (ILR-1), and activating transcription factor 2, while central proteins in HFpEF were catenin beta-1 and integrin subunit beta-2. HFrEF was related to DNA binding transcription factor activity, regulation of nitric oxide biosynthesis, and cellular protein metabolism. However, HFpEF was related to cytokine response, extracellular matrix organization, and inflammation. In addition to the above, in the Nadar et al. study [120], markers of inflammation such as high-sensitivity C-reactive protein (hsCRP), ST2, and cystatin C (CysC) levels and markers of myocardial fibrosis such as galectin-3 were identified to be increased in HFpEF patients.

In the Bielecka-Dabrowa et al. study [128], biomarkers with different pathophysiological backgrounds (NT-proBNP, CT-1, TGF-β, and CysC) gave additive prognostic value for incident HF in hypertensive patients compared to NT-proBNP alone. Michalska-Kasiczak et al. [129] also noted that a single biomarker may not be sufficient in clinical practice in a heterogeneous group of HF patients. They suggested that is necessary to use a biomarker panel. Biomarker profiles of patients with HFmrEF, HFpEF, and HFrEF are different. The biomarkers in HFpEF are mainly based on inflammation, while in HFrEF, they are more cardiac stretch based, and in HFmrEF, they are related to both inflammation and cardiac stretch. Biomarkers associated with inflammation and heart remodeling are predictive in HFmrEF and HFpEF but not in HFrEF. These data could have important therapeutic consequences for the group of HF patients and suggest that it is necessary to use a biomarker panel [120].

According to recent news, neutrophil gelatinase-associated lipocalin (NGAL), a marker of renal injury, seems to be also a good factor in the diagnosis and prognostic prediction in HF [130].

Different miRNAs (miR423-5p, miR320a, and miR22) could be increased in patients with HF [131]. A recent study suggested that miR423-5p could be the best potential biomarker [132].

## Previous HF hospitalization

Hospitalization for HF within the last year has been significant risk factor of subsequent hospitalizations. The results of the QUALIFY survey showed that 30.4% of the patients had a history of at least two HF hospitalizations [133]. In the ESC-HF Pilot study, 57% of the HF patients had a history of previous hospitalizations [134] and, additionally, 24.75% of them were rehospitalized in a 1-year follow-up [135].

## Prognostic factors in different multivariate predictor models

**Fig. 3:**
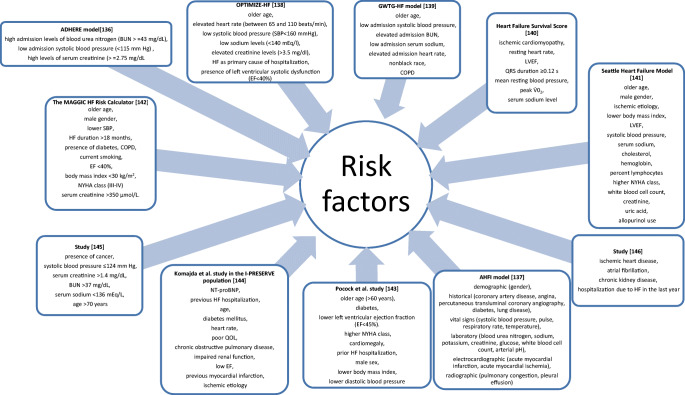
Selected risk models for the assessment of prognosis in heart failure

Different risk models were constructed to assess different clinical endpoints in the HF population [17]. Figure 3 provides a list of studies that have identified many prognostic values in HF (Fig. 3).

## Conclusion

All of the presented models have shown only moderate probability in predicting death in HF [17]. Moreover, while their effects appear to be acceptable at the population level, they do not sufficiently predict outcome for an individual patient [147]. As we can see, there are also numerous parameters that may be used in clinical practice and should be used in order to determine the overall risk of our patients as accurately as possible.

Cited studies allow for the isolation of variables included in the models more often than others: sex, age, SBP, HR, NYHA class, LVEF, BUN level, serum creatinine, and sodium concentration. Other strong prognostic factors of mortality in HF, consistently reported in different models, include BNP/NT-proBNP concentration, weight or body mass index, and diabetes mellitus [17, 135–147]. Nevertheless, there is no possibility at the moment to assess and monitor HF with a single parameter or a simple scale that would apply to the whole population of patients.

Despite the identification of many markers and models of poor prognosis, clinical decisions and guidelines in HF are still based mainly on several basic parameters, such as the presence of HF symptoms (NYHA class), LVEF, and the duration and morphology of the QRS complex [17]. But considering the cited works, all potential tools for assessing the risk of HF patients should be used if possible.
